# Graphene oxide loaded with tumor-targeted peptide and anti-cancer drugs for cancer target therapy

**DOI:** 10.1038/s41598-021-81218-3

**Published:** 2021-01-18

**Authors:** Ran Li, Yimei Wang, Jie Du, Xiangyu Wang, Ailin Duan, Ruifang Gao, Junyu Liu, Bing Li

**Affiliations:** 1 Shanxi Medical University School and Hospital of Stomatology, Taiyuan, 030001 China; 2 Shanxi Province Key Laboratory of Oral Diseases Prevention and New Materials, Taiyuan, 030001 Shanxi China

**Keywords:** Cancer therapy, Cancer, Oral diseases, Nanoscience and technology, Graphene, Nanomedicine

## Abstract

In the present work, we constructed nanoscale graphene oxide (NGO) as a drug nanocarrier to improve the process of tumor-targeted drug releases, promote cellular uptake and accumulation of chemotherapy drugs in tumor tissues, and reduce the toxic effects of chemotherapy drugs on normal cells. Hence, great stability was obtained in the biological solution. Moreover, we designed an effective nanoparticle system for the doxorubicin (DOX) delivery targeting the oral squamous cell carcinoma (OSCC) by mediating the HN-1 (TSPLNIHNGQKL) through hydrogen and π–π bonds. DOX@NGO-PEG-HN-1 showed significantly higher cellular uptakes and cytotoxicity in OSCC cells (CAL-27 and SCC-25), compared to free DOX. Moreover, HN-1 showed considerable tumor-targeting and competition inhibition phenomenon. As we expected, the nanocarrier showed pH-responsive drug release. In total, our study represented a good technique to construct OSCC-targeted delivery of nanoparticles and improve the anticancer medicines’ efficiency.

## Introduction

In recent years, oral squamous cell carcinoma (OSCC) has had a high incidence. Surgery, chemotherapy, and radiation therapy are commonly utilized treatments currently^1^. Recently, traditional surgical treatment accompanied by chemotherapy is the main therapeutic approach^2^. In addition to the surgery risk, it often causes greater damage to the patient's salivary glands, causing dry mouth, language dysfunction and feeding disorder. The postoperative life quality of patients has been greatly reduced by various factors causing many negative effects on patients' psychology^3^. Generally, clinical chemotherapy drugs have lower cellular uptake as well as certain toxicity and side effect on normal cells, which seriously limits their clinical application^4,5^. Nano drug delivery system has attracted the attention of many researchers^6,7^. GO offers several brilliant characteristics in this regard, including large specific surface^8^, high drug loading rate^9^, pH-responsiveness^10,11^, and EPR effect^12^. Hence, they become appropriate for integration in a range of delivery systems for cancer treatment^13–16^. EPR effect is the enhanced permeability and retention effect is that the small size of nanocarriers (20–200 nm) penetrate through the leaky tumor vessels and finally gather in the tumor interstitial space^17,18^. Some researchers indicated that graphene oxide can be used as a nanocarrier for loading and delivery^19–21^ of commonly used anticancer drugs such as SN-38^22^, doxorubicin, camptothecin, and methotrexate^23,24^. Moreover, a higher drug loading rate of 200% was reported for the DOX/NGO^25^. Though the stability of nanoscale graphene oxide (NGO) in an aqueous solution is not ideal after loading with hydrophobic drugs^26,27^, some improvements are required.

The GO’s surfaces and edges have abundant oxygen-containing functional groups conducive to surface functionalization^28,29^. To improve the stability in an aqueous solution and its biocompatibility, the NGO was functionalized frequently by polyethylene glycol (PEG)^30^. It generates an excellent biomaterial for biomedical applications especially for drug delivery^9,31^ while reducing their nonspecific absorption to biological molecules and cells^32,33^.

In this study, we synthesized an NGO-PEG nanocarrier and examined its cytotoxicity effects on CAL-27, SCC-25, and human normal oral keratinocytes (HOK). Based on the previous studies, antibodies^34–36^, peptides^37,38^, and aptamers^39,40^ are always utilized to surface-modify NGOs as the targeting substances. The HN-1 peptide (TSPLNIHNGQKL) is a 12-amino acid peptide recently isolated from the phage display library^41^. It was reported that this astonishingly small peptide was specific to the OSCC cells and able to penetrate the tumor tissue^42^. Since the therapeutic effect of DOX@NGO-PEG is limited in drug delivery by the lack of active tumor targeting and poor cancer cell internalization, we introduced HN-1 into the drug delivery system for the first time. Our study indicated that DOX@NGO-PEG-HN-1 exhibits tumor-targeting characteristics and pH-responsiveness drug release characteristics.

## Results

### Preparation and characterization of DOX@NGO-PEG-HN-1

**Figure 1:**
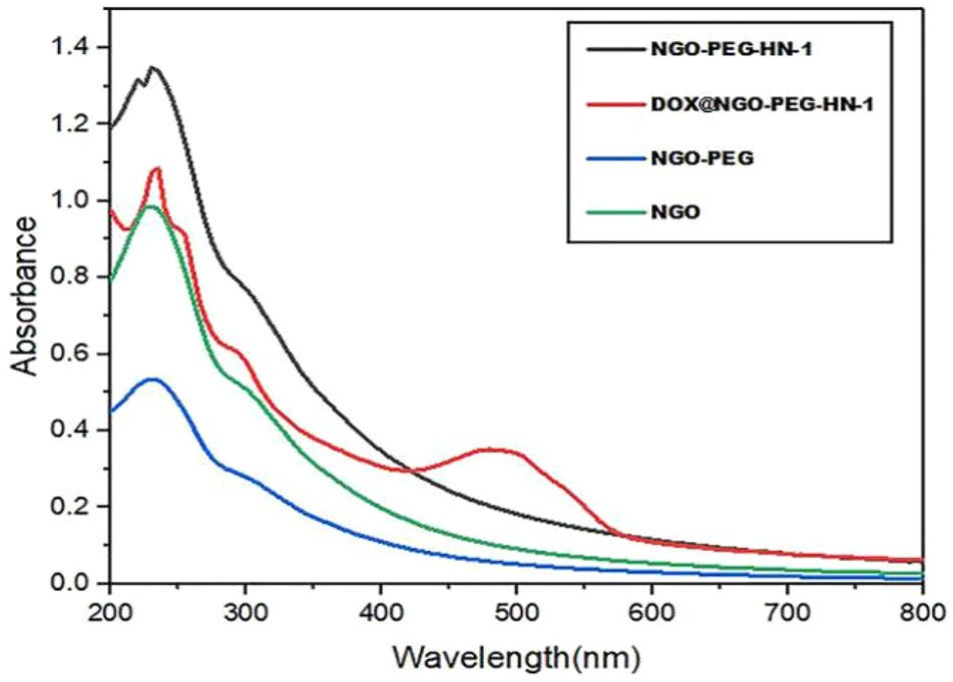
The UV–Vis spectra of NGO, NGO-PEG, NGO-PEG-HN-1 and DOX@NGO-PEG-HN-1.

UV results (Fig. 1) indicate that the NGO possesses a maximum absorption peak at 230 nm, which is the characteristic absorption peaks of C=O. NGO-PEG has a maximum absorption peak at 250 nm after PEG modification. This is caused by the gradual reduction of the NGO, and the absorption peak moving toward 250 nm. The peptide HN-1 has a clear UV absorption peak at 220 nm, and the characteristic peak of HN-1 appears at 220 nm for NGO-PEG-HN-1, indicating the successful binding of HN-1 and NGO-PEG. DOX has an obvious absorption peak at 494 nm, and DOX @ NGO-PEG-HN-1 possesses an obvious absorption peak at 494 nm. Hence, it can be judged that DOX is successfully combined with NGO.

**Figure 2:**
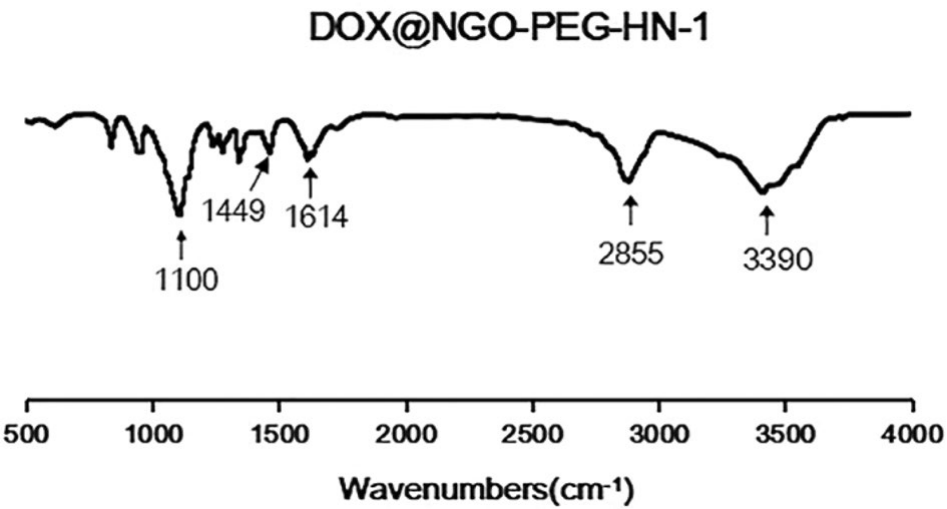
The FTIR spectra of DOX@NGO-PEG-HN-1.

In the FTIR spectrum of DOX@NGO-PEG-HN-1 (Fig. 2), the absorption peak at 3390 cm^−1^ represents the stretching vibration of hydroxyl (-OH), and the strong absorption peak at 1100 cm^−1^ denotes the stretching vibration of C–O–C. Hence, it is indicated that PEG was grafted successfully to NGO. A new peak was observed at 1409 cm^−1^. The changes in these chemical bond vibration peaks approved the bond formations of NGO-PEG with HN-1 peptides, through hydrogen bond and π–π bond interactions. The absorption peaks at 1614 and 2855 cm^−1^ are resultant by the stretching vibration peaks of –CO–NH– bond and –NH– bond respectively revealing that DOX was successfully conjugated.

**Figure 3:**
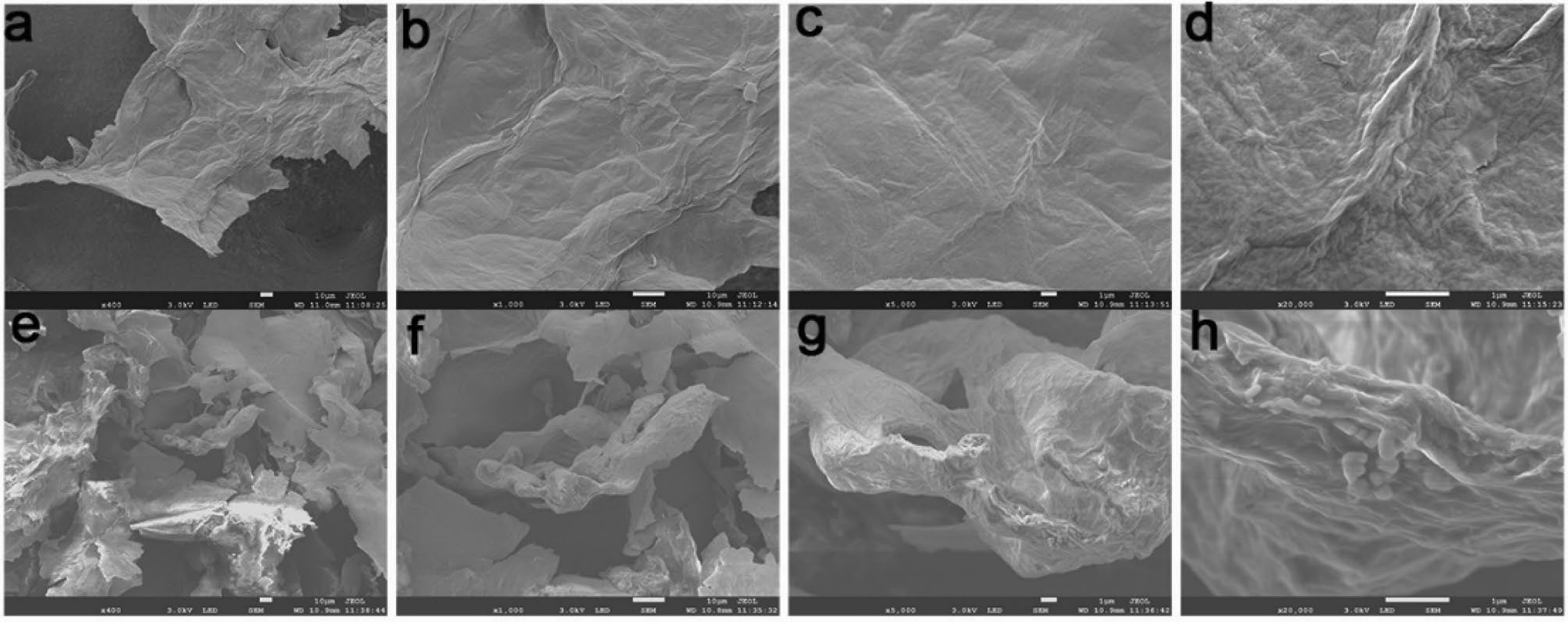
The scanning electron microscope images of (**a**–**d**) NGO, (**e**–**h**) DOX@NGO-PEG-HN-1.

The morphology and surface properties of NGO, DOX@NGO-PEG-HN-1 were investigated using a scanning electron microscope (SU8020, Hitachi, Japan). According to the represented SEM images, NGO possess a lamellar-like structure (Fig. 3a–d). However, DOX@NGO-PEG-HN-1 represents obvious wrinkles and curls (Fig. 3e–h) different from NGO. Moreover, the structure is approximately porous as a result of the *π*–*π* stacking bond between the aromatic ring of DOX and graphene oxide plates. Furthermore, there are small particles on the surface (Fig. 3h), that are HN-1 peptides. The GO layer spacing was incremented by the introduction of PEG and HN-1.

**Figure 4:**
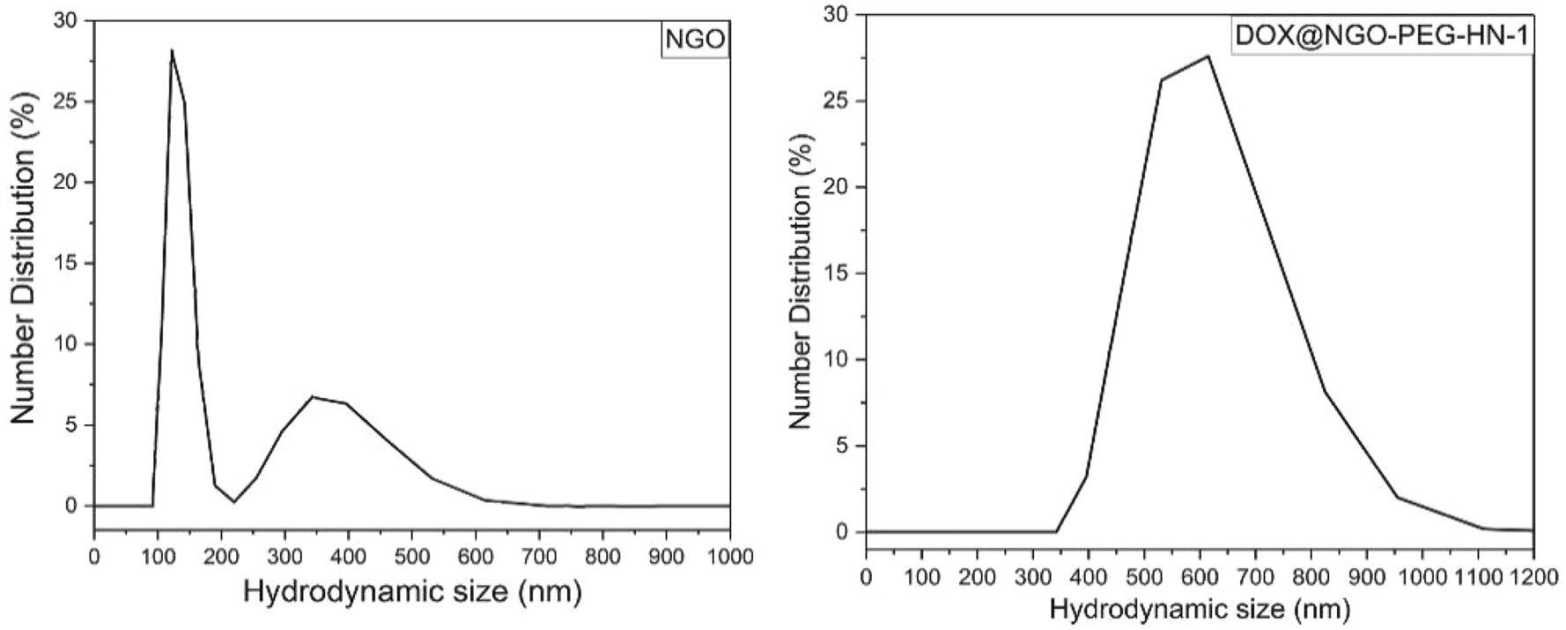
The particle size distribution: number distribution of NGO and DOX@NGO-PEG-HN-1.

The size of NGO and DOX@NGO-PEG-HN-1 was analyzed with the dynamic light scattering method. First, the sample dispersed in water was sonicated for 10 min and then was analyzed with the Zetasizer. It was found (Fig. 4) that the NGO had narrow distribution within the range of 100–200 nm with a median value of 150 nm. About 5% of NGO were distributed in the range of 200-600 nm with the median value of 380 nm. This was due to the NGO itself was not uniform. However, the nanocomposite, DOX@NGO-PEG-HN-1 had a wider distribution, within the range of 350 to 950 nm.

### The result of loading capacity of DOX on NGO

**Figure 5:**
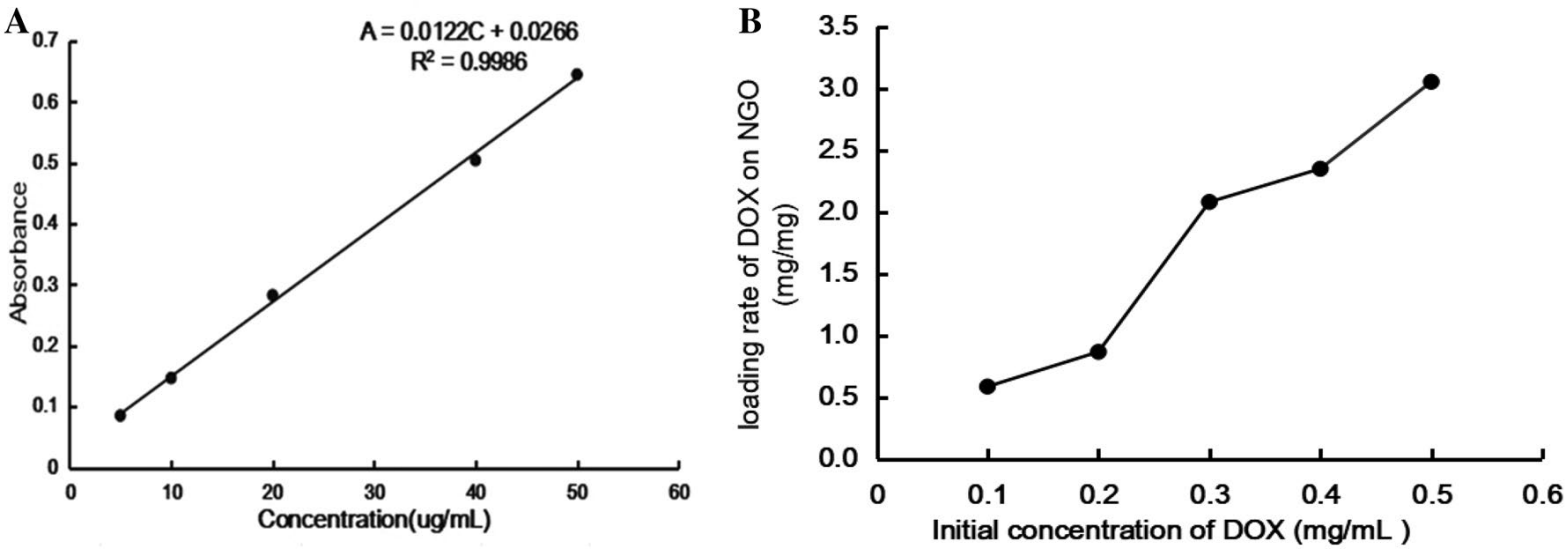
(**A**) The standard curve of DOX. (**B**) The loading rate of DOX on NGO in different concentration of DOX.

The UV–visible absorbance of the DOX was tested at 480 nm and then draw the standard curve (Fig. 5A). It can be seen that the linear regression equation of DOX was: A = 0.0122C + 0.0266 (R^2^ = 0.9986) where A was the UV–visible absorbance of DOX at 480 nm, C was the concentration of DOX (μg/mL), and R^2^ = 0.9986, indicating that there was a good linear relationship, and the standard curve was credible.

The loading rate of DOX on NGO was calculated by weight of the loaded DOX/weight of the NGO. As shown in Fig. 5B, When the initial concentration of DOX was 0.1 mg/mL, the value of DOX/NGO was 0.58 mg/mg. As the initial concentration of DOX increases, the value of DOX/NGO also increases. And, this value was already as high as 3.05 mg/mg when the DOX concentration was 0.5 mg/mL. This showed that the NGO was indeed apromising drug carrier materials.

### The results of DOX loading and in vitro pH-responsive drug release

**Figure 6:**
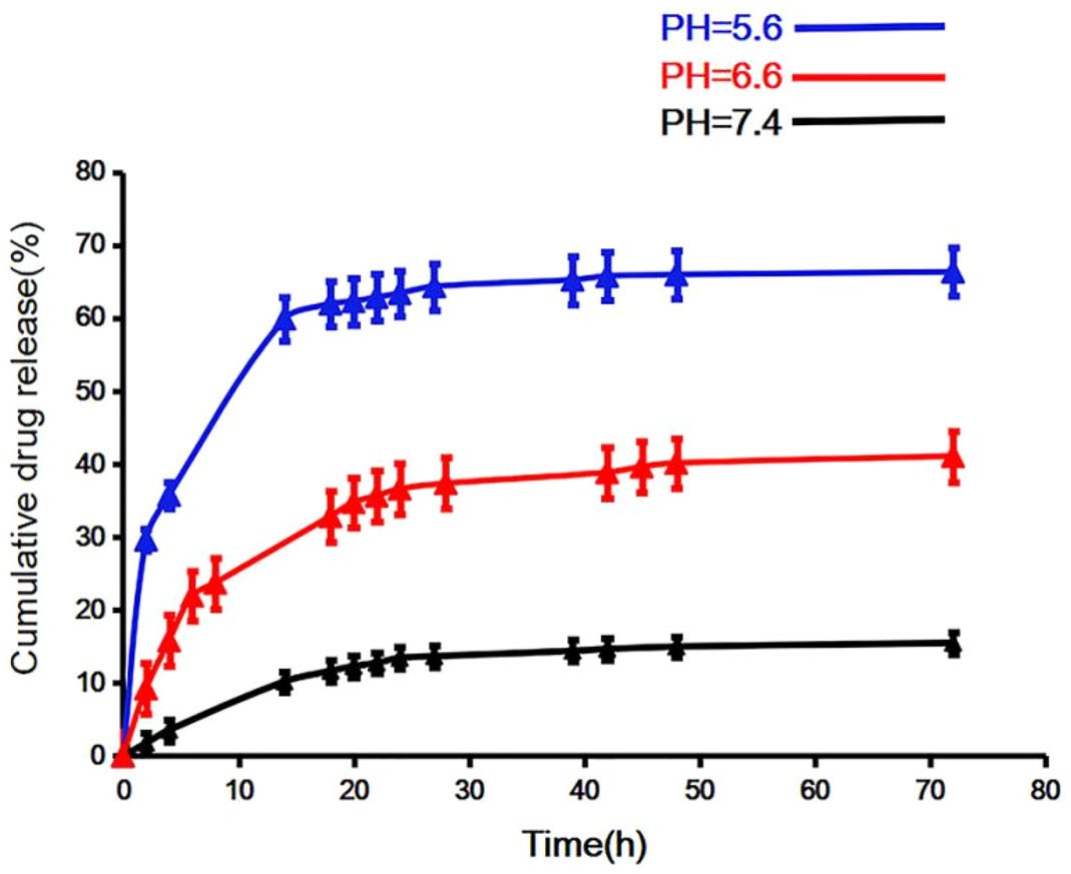
In vitro drug release profiles of DOX@NGO-PEG-HN-1 at different pHs environment.

Comparing the drug release at three different pH buffers, it is observed (Fig. 6) that the drug release amount and release rate at pH 5.6 are higher compared to pH 6.6 and pH 7.4 at the same time interval. The reason is that the DOX and the carrier are bound to each other through π–π interaction and hydrogen bonding interaction, moreover, the acidic environment can weaken the force of hydrogen bonds and π–π interaction^43^, while releasing DOX from the carrier. In an acidic environment, the drug release rate reaches 70%, however, at pH 6.6, the release rate reaches only 30%, and it is even less at pH 7.4. In the first 24 h, the drug release rate was faster, though it was slower after the first 24 h. In the tumor tissue, it is a slightly acidic environment relative to normal tissue. The toxicity can be reduced by this feature of the drug carrier to normal tissues to some extent.

### The results of the stability experiment

**Figure 7:**
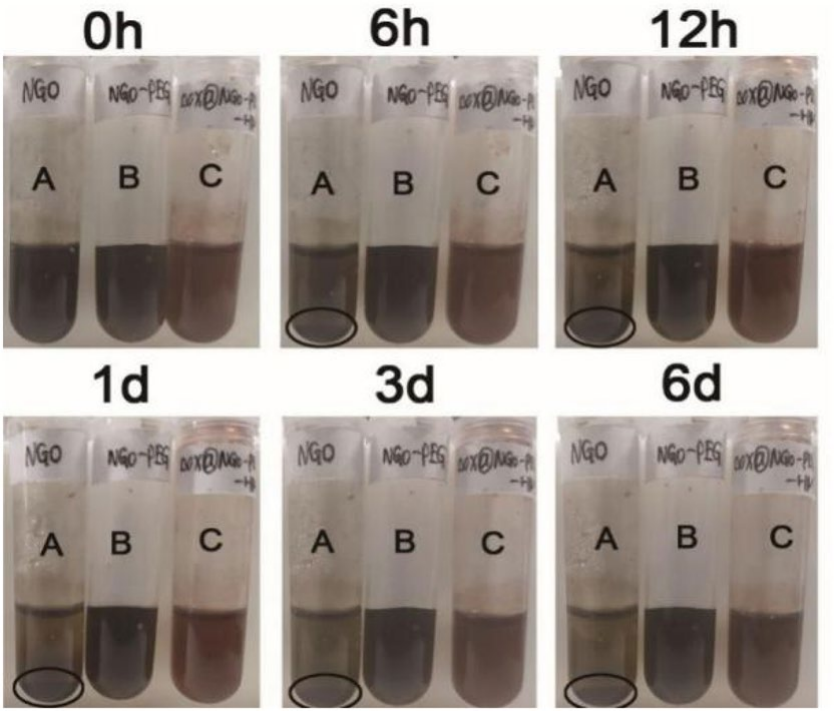
The results of the stability experiment of NGO, NGO-PEG, DOX@NGO-PEG-HN-1 in water (1 mg/mL). (A) NGO, (B) NGO-PEG, (C) DOX@NGO-PEG-HN-1.

Owing to the poor hydrophilicity of the NGO, we utilized PEG to enhance the stability of the NGO. According to the picture, for NGO, precipitation appeared after standing 6 h, moreover, obvious sediments can be seen after 1 day in water (Fig. 7A). By contrast, the NGO-PEG and DOX@NGO-PEG-HN-1 were evenly dispersed and stable after 6 days (Fig. 7B,C). It exactly proved that the PEG conjunction could enhance the biocompatibility and stability of NGO.

**Figure 8:**
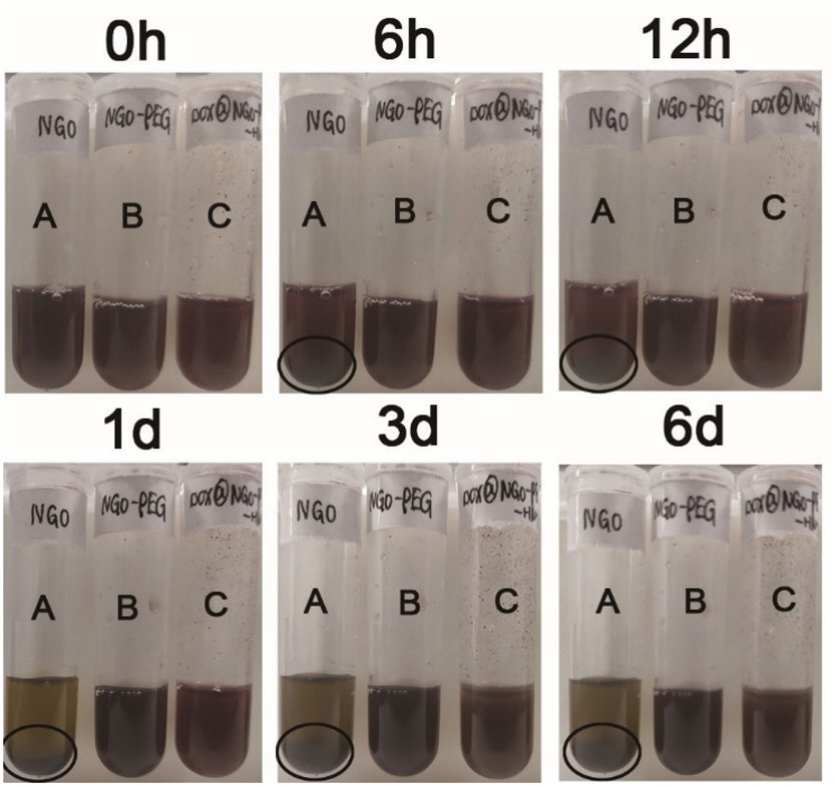
The results of the stability experiment of NGO, NGO-PEG, DOX@NGO-PEG-HN-1 in10% FBS (1 mg/mL). (A) NGO, (B) NGO-PEG, (C) DOX@NGO-PEG-HN-1.

To simulate the blood environment in vivo, we observed the stability of the nanocomposite in a 10% FBS experimental condition. According to the Fig. 8, after 6 h standing there were few sediments, and a huge deal of precipitation was observed after 1 day for NGO (Fig. 8A), which is consistent with the result in water. Compared to NGO, NGO-PEG, and DOX@NGO-PEG-HN-1 (Fig. 8B,C) have been stably dispersed in the 10% FBS condition without precipitation. Thus, we can conclude that the stability of the PEG-modified nanocomposite is enhanced.

### Results of cell uptake

**Figure 9:**
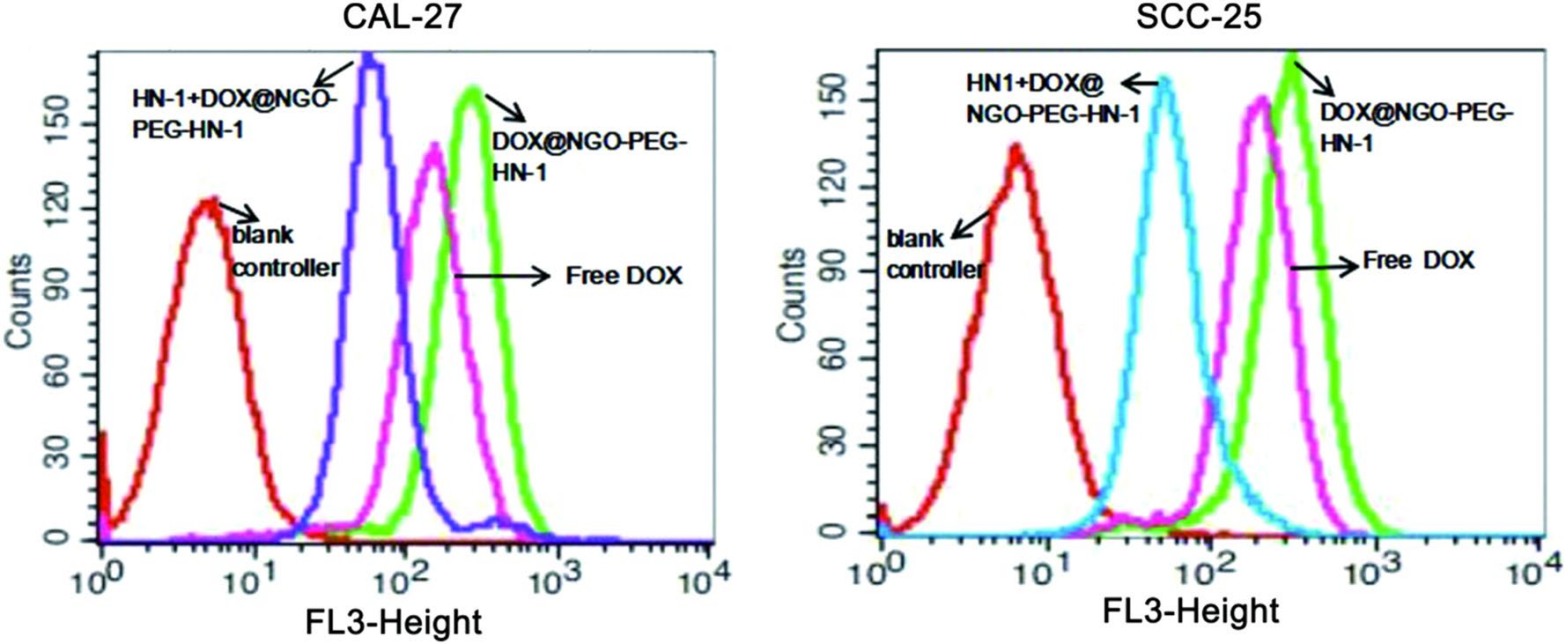
CAL-27 and SCC-25 cell uptake ration of DOX when incubated with DOX@NGO-PEG-HN-1, free DOX or HN-1 + DOX@NGO-PEG-HN-1.

The results of cell uptake rationed by flow cytometry are shown in Fig. 9. Larger quantities of DOX were internalized by SCC-25 and CAL-27 in the DOX@NGO-PEG-HN-1 group. This more objectively illustrates that the nano-drug loading system can accelerate the drug internalization. However, the cellular uptake ratios decreased in groups incubated with HN-1 in advance in comparison to DOX@NGO-PEG-HN-1. The reason is that competition inhibition occurs after incubation with HN-1, and the internalization of the drug was inhibited to a certain extent. More objective and accurate description of our nanocarriers can help DOX to more accurately and quickly internalize into tumor cells, thereby improving efficacy.

### The results of intracellular localization of DOX@NGO-PEG-HN-1-FITC

**Figure 10:**
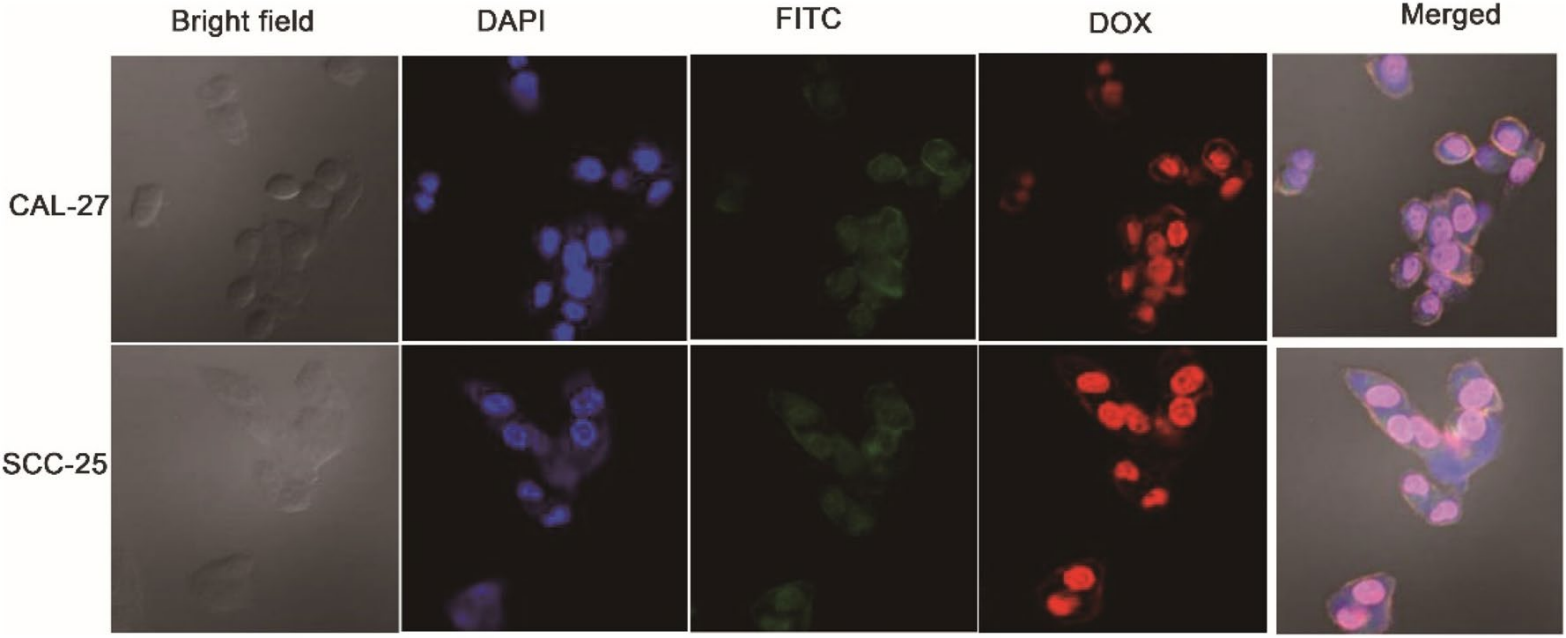
FITC, DAPI, and DOX images of CAL-27 and SCC-25 cells treated with DOX@NGO-PEG-HN-1-FITC.

To further comprehend the internalization process of the nanocomposite, we purchased an HN-1 peptide labeled with FITC and used it to prepare DOX@NGO-PEG-HN-1-FITC. As a fluorescein marker, FITC is extensively utilized in drug research and cell biology^44^. Drugs can be internalized by cells, which is an essential condition to achieve treatment. In this test, the nano drug-loading system was incubated with CAL-27, SCC-25 for 4 h, and the results of drug internalization were observed with a laser confocal microscope. According to Fig. 10, compared to the staining of the nuclei by DAPI, the localization of FITC and DOX were approximately consistent with the DAPI image, which was both at the nucleus position. This phenomenon firmly verified that nanocarriers can obtain DOX’s internalization process.

### In vitro cell cytotoxicity

**Figure 11:**
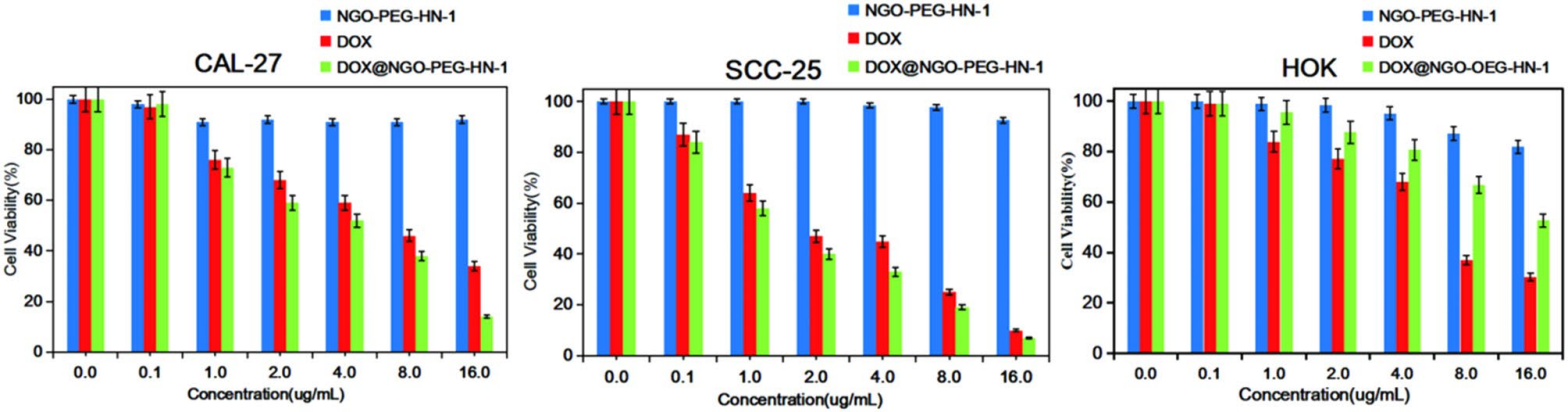
The Cell Viability of CAL-27 SCC-25 and HOK co-cultured with DOX, NGO-PEG-HN-1, and DOX@NGO-PEG-HN-1 for 24 h.

Based on Fig. 11, the cell viability of the NGO-PEG-HN-1 group is nearly 100% in three kinds of cells. It is indicated that the NGO-PEG is relatively safe and non-toxic to the cells, which is consistent with the previous reports^45–49^. For CAL-27 and SCC-25 cells, it was not difficult to see from Fig. 11 that DOX@NGO-PEG-HN-1 had a greater cytotoxic effect than free DOX. Considering that the drug will not be completely released in a limited time, the amount of the drug that DOX@NGO-PEG-HN-1 actually acted on the cells was much lower than the amount of free DOX in the same group. But the DOX@NGO-PEG-HN-1 group still has lower cell viability, this is because that the nanocarrier can accelerate the internalization of the drug, and the DOX acts on the nucleus faster, making the drugs more efficiently. For HOK cells, the DOX group’s cell viability is significantly lower compared to DOX @ NGO-PEG-HN-1. Since HN-1 is a targeting peptide for squamous cell carcinoma and normal epithelial cells HOK, the targeted internalization of drugs is not accelerated by the nanocarrier NGO-PEG-HN-1, and the pH environment of normal cells is not conducive to drug release. Furthermore, HN-1 possesses strong capabilities for targeting and penetrating OSCC cells^57^. Observing the effects of DOX and DOX @ NGO-PEG-HN-1 on CAL-27 and SCC-25 cells, it was revealed that SCC-25 is more sensitive to the drugs’ effects.

## Conclusions

In conclusion, we successfully made a pH-sensitive cancer-combination treatment system DOX@NGO-PEG-HN-1. Achieving the DOX@NGO-PEG-HN-1 at tumor tissue, the DOX can be quickly released from the system as a result of the weakened hydrogen bonding interaction and π–π conjugation between GO and DOX. The experimental findings of the cytotoxicity prove that the DOX@NGO-PEG-HN-1 can be simply absorbed by tumor cells and exhibited a collaboration effect. This drug-loading system has great application potential in treating tumors.

## Discussion

There are reports in the literature regarding the photothermal effect of nanographene oxide. Indeed, under certain near-infrared irradiation, light energy can be converted into heat energy locally increasing the temperature of tumor tissue, thereby killing tumor cells. The nanocarriers’ photothermal features can be further explored and the metabolism and targeting of drugs can be further investigated in vivo.

Some researchers have studied the toxicity in vivo*.* Organ tissue section analysis, blood routine, and biochemical indicators indicated that NGO-PEG had no significant damage to liver and kidney functions in mice^50^. Specifically, the toxicity of our drugs in vivo requires further experiments.

As for the targeting peptide HN-1 of oral squamous cell carcinoma, its targeted internalization mechanism is not well-known at present. Then, we can further explore the molecular mechanism of HN-1 targeting into oral squamous cell carcinoma cells. Novel ideas are provided by an in-depth exploration of its signal pathways for treating oral squamous cell carcinoma from the molecular level.

## Materials and methods

### Chemistry

Graphene oxide (GO) was brought from Nanjing XFNANO Materials. Polyethylene glycol (PEG4000), NaHCO_3_, and EDTA were provided by Shanghai Aladdin Biochemical Technology Co., Ltd. Dialysis bags (MWCO:8000-14000) was purchased from Beijing Soledad Biotechnology Co., Ltd. Hangzhou peptide was used to synthesize HN-1polypeptide and FITC-HN-1 in biochemical co., Ltd. Doxorubicin hydrochloride (DOX) was acquired from Dalian meilun biotechnology co., Ltd. Dulbecco’s Modified Eagle’s Medium (DMEM), Dulbecco’s phosphate buffered saline (PBS) and fetal bovine serum (FBS) were purchased from BOSTER, Wuhan, China.

### Preparing graphene oxide (NGO-PEG)

Dispersed carboxylated-GO in the double-distilled water, added terminally aminated PEG4000 (NH_2_-PEG4000-NH_2_) while sonicating for 30 min, and then 40 mmol/L EDC-HCL (1-(3-dimethylaminopropyl)-3-ethyl carbodiimide hydrochloride) was added as a catalyst and stirred at room temperature overnight^51,52^. The solution was placed in a dialysis bag (MWCO = 100kD) after the reaction, and no reacting parts were removed by dialysis with pure water for 4 days in darkness to achieve the desired NGO-PEG.

### Preparation of NGO-PEG-HN-1 and NGO-PEG-HN-1-FITC

Adding 10 mL of NGO-PEG and HN-1-FITC or HN-1 to the double-distilled water, it was stirred violently in darkness for 24 h at room temperature. Then, the free HN-1 or FITC-HN-1 was eliminated through dialysis with pure water in darkness for 7 days^53^.

### Preparation of DOX@NGO-PEG-HN-1 and DOX@NGO-PEG-HN-1-FITC

Doxorubicin (DOX) was added into NGO-PEG-HN-1 or NGO-PEG-HN-1-FITC solution and then stirred at room temperature overnight. DOX would be loaded onto the NGO-PEG-HN-1 carriers through hydrogen bond and π–π bond^54–57^. To remove free DOX, the mixture was centrifuged at 15,000 rpm for 5 min, and the supernatant was collected. The fluorescence intensity of the supernatant was measured via fluorescence spectrophotometer. The drug loading efficiency (DLE) of DOX@NGO-PEG-HN-1 was assessed as follows^58^:

$$ {\text{DLE}}\left( \% \right) = \frac{{{\text{m}}_{{{\text{total}}}} - {\text{m}}_{{{\text{unloaded}}}} }}{{{\text{m}}_{{{\text{total}}}} }} \times 100\% . $$

### The loading capacity experiment of DOX on NGO

Added 6 mg of DOX into 6 mL of the distilled water, and dispersed it with ultrasound until it was totally dissolved. Then the solution was diluted gradually to obtain the following concentration solutions: 5 ug/mL, 10 ug/mL, 20 ug/mL, 40 ug/mL, 50 ug/mL. Lastly, UV–Vis spectrophotometer (UV–Vis) was used to measure the absorbance of different concentrations of DOX at a wavelength of 480 nm. Use the concentration of DOX as the abscissa and the absorbance of DOX as the ordinate to draw a standard curve.

Added different concentrations of DOX (0.1 mg/mL, 0.2 mg/mL, 0.3 mg/mL, 0.4 mg/mL, 0.5 mg/mL) solutions to 1 mL NGO (0.1 mg/mL) PBS solution, and ultrasound 0.5 h, then stirred overnight at room temperature, and finally centrifuged the solution at high speed (14000 rpm, 0.5 h), collected the supernatant, and measured the absorbance at 480 nm with an UV spectrophotometer. Loading content (LC%) = (Weight of loaded DOX)/(Weight of NGO).

### In vitro pH-dependent drug release

Equally 6 mL NGO-PEG-HN-1-DOX was distributed into three dialysis bags. The dialysis bags were immersed in 100 mL of different pH buffer solution (5.6, 6.6, and 7.4), then placed the solution on the Shaker at 37 °C (SHZ-82A) with gentle shaking (150 rpm). Withdrawing 2 mL of dialysate within the specified time intervals, 2 mL corresponding fresh buffer solution was added into each sample. The quantity of released DOX was detected by a fluorescence spectrophotometer at a set time interval of 480 nm (λex = 488 nm; slit width = 5 nm). The cumulative drug release is calculated as follows:

$$ {\text{Cumulative}}\;{\text{release}}\,\left( \% \right) = \frac{{2 \times \sum\nolimits_{i = 1}^{n - 1} {Ci + 100 \times Cn} }}{{{\text{weight}}\,{\text{of}}\,{\text{drug}}\,{\text{in}}\,{\text{the}}\,{\text{DOX@NGO - PEG - HN - }}1}} \times 100\% . $$

### Stability experiment

To enhance the biocompatibility of the NGO complex, we used PEG to modify the complex. The concentrations of NGO, NGO-PEG, DOX@NGO-PEG-HN-1 were 1.0 mg/mL in water and serum-containing solution. Then, we evaluated its stability by observing the existence of precipitation over a 6-day standing still.

### Cell uptake of DOX@NGO-PEG-HN-1

The experiment of cellular uptake of graphene oxide materials in vitro was conducted on CAL-27 and SCC-25 cells. First, SCC-25 and CAL-27 cells in the logarithmic growth phase were taken and planted into six Petri dishes at a density of 1X10^6^ per well, and 2500 μL of complete culture medium was added. The cells were incubated for 24 h in a 5% CO_2_ incubator at 37 ℃. Then, free DOX (concentration 5 μg/mL), DOX @ NGO-PEG-HN-1 was dispersed in a complete culture medium. As for the rest two Petri dishes, HN-1 was co-cultured 1 h with CAL-27 and SCC-25 cells respectively before adding DOX@NGO-PEG-HN-1 in the complete culture medium. After co-cultivation for 2 h, the medium was discarded and the cells were rinsed with sterile ice PBS several times. Then, the cells were digested with trypsin and collected in PBS, and the DOX incorporation rate was detected by flow cytometry.

### Intracellular localization of DOX@NGO-PEG-HN-1-FITC

The experiment of intracellular localization of DOX@NGO-PEG-HN-1-FITC was conducted on OSCC cells (CAL-27, SCC-25). First, cells at a density of 5 × 10^4^ were seeded into confocal dishes and incubated for 24 h. Then, DOX@NGO-PEG-HN-1-FITC was added to each dish and incubated for 4 h. PBS was used then to rinse the cells three times, and cells were then fixed in 4% paraformaldehyde (PFA) for 0.5 h. Ultimately, the cells were rewashed with PBS three times and then stained with DAPI for 10 min. Confocal microscopy (Leica TCS SP2, Mannheim, Germany) was used to track the localization of DOX@NGO-PEG-HN-1-FITC on CAL-27 and SCC-25 cells.

### In vitro cytotoxicity test

The drug carrier materials’ cytotoxicity (DOX, NGO-PEG-HN-1, DOX@NGO-PEG-HN-1) was examined by CCK8 assay on human tongue squamous carcinoma CAL-27, SCC-25 cells, and human normal oral keratinocytes (HOK) as a negative control group. Here, six different drug concentrations were adjusted (0.1, 1, 2, 4, 8, 16 μg/mL) for three types of materials (DOX, NGO-PEG-HN-1, DOX@NGO-PEG-HN-1). Considering that the drug loading rate is 44.6%, we synthesized DOX@NGO-PEG-HN-1 proportionally to ensure that the amount of DOX in the DOX@NGO-PEG-HN-1 group and the free DOX group was the same. First, the cells were seeded in a 96-well plate at a density of 4 × 10^3^ per well with 100 μL DMEM medium comprising 10% FBS and 1% chain penicillin and incubated for 24 h in a cell incubator (37℃, 5% CO_2_). Subsequently, the drug carrier materials were added to each well and incubated for another 24 h at the same condition. Ultimately, the medium and drug carrier materials in the 96-well plate were aspirated, and fresh medium and 10 μL of CCK8 reagent were added. The absorbance was measured with a microplate reader at 450 nm followed by incubation for 2 h. The cell vitality was calculated as follow:

$$ {\text{Cell}}\;{\text{viability}} = \frac{{{\text{OD}}_{{{\text{treated}}}} - {\text{OD}}_{{{\text{blank}}}} }}{{{\text{OD}}_{{{\text{control}}}} - {\text{OD}}_{{{\text{blank}}}} }} $$

OD_treated_, OD_control_, OD_blank_ are the absorbance values of the sample wells. The final OD value is obtained by mean ± standard deviation (SD) of the values of the six independent parallel specimens.
